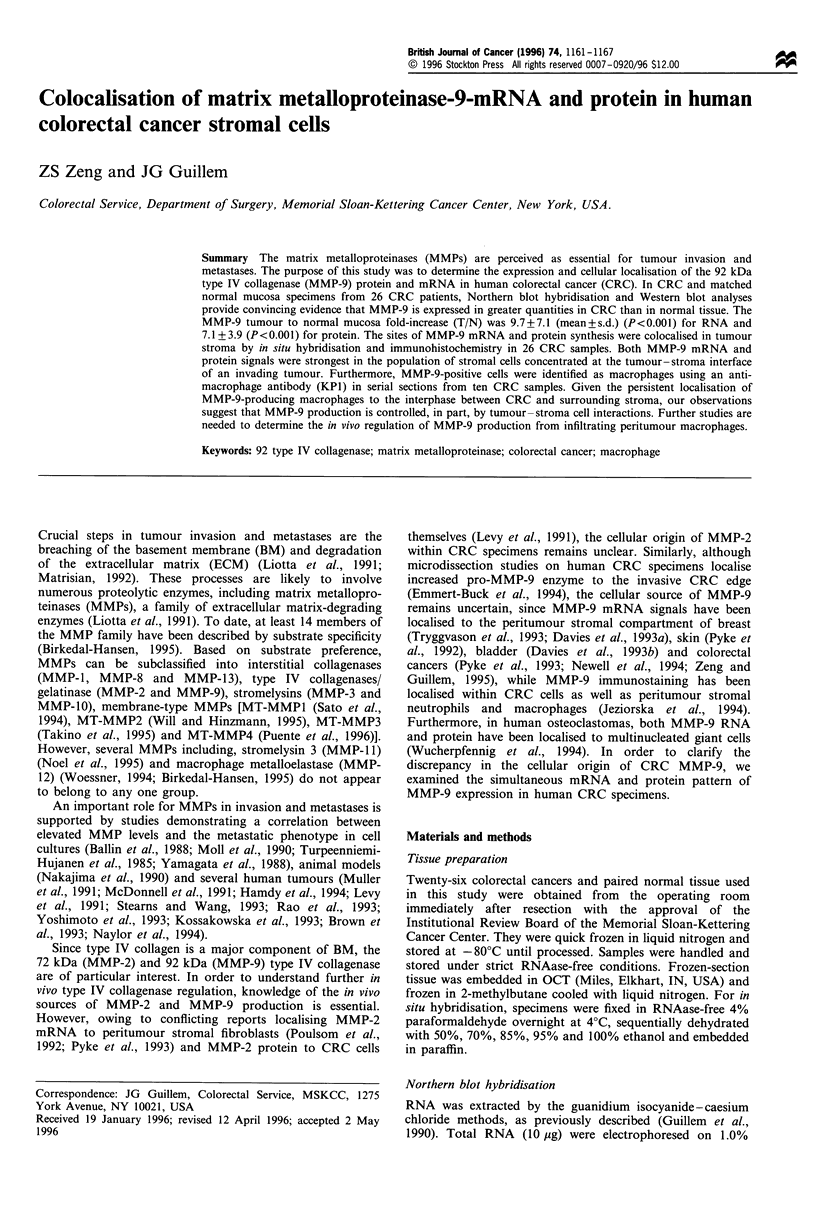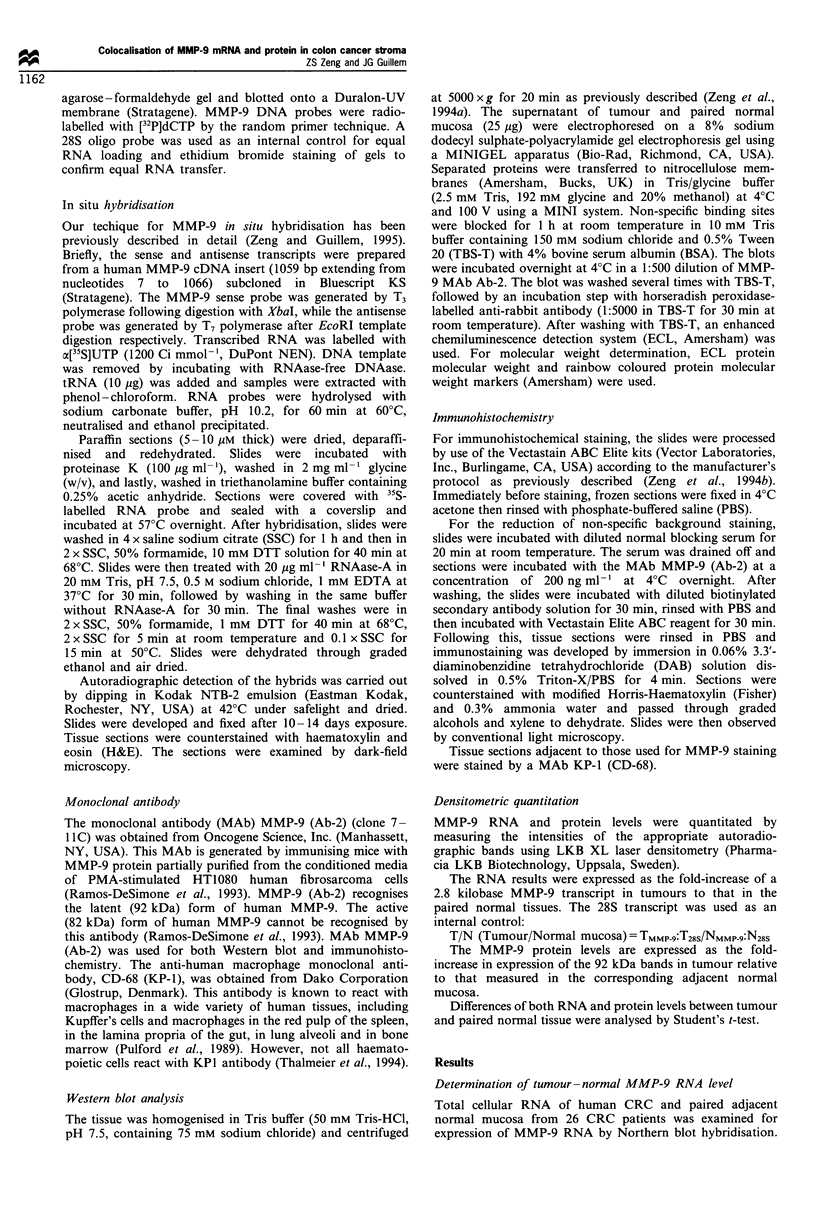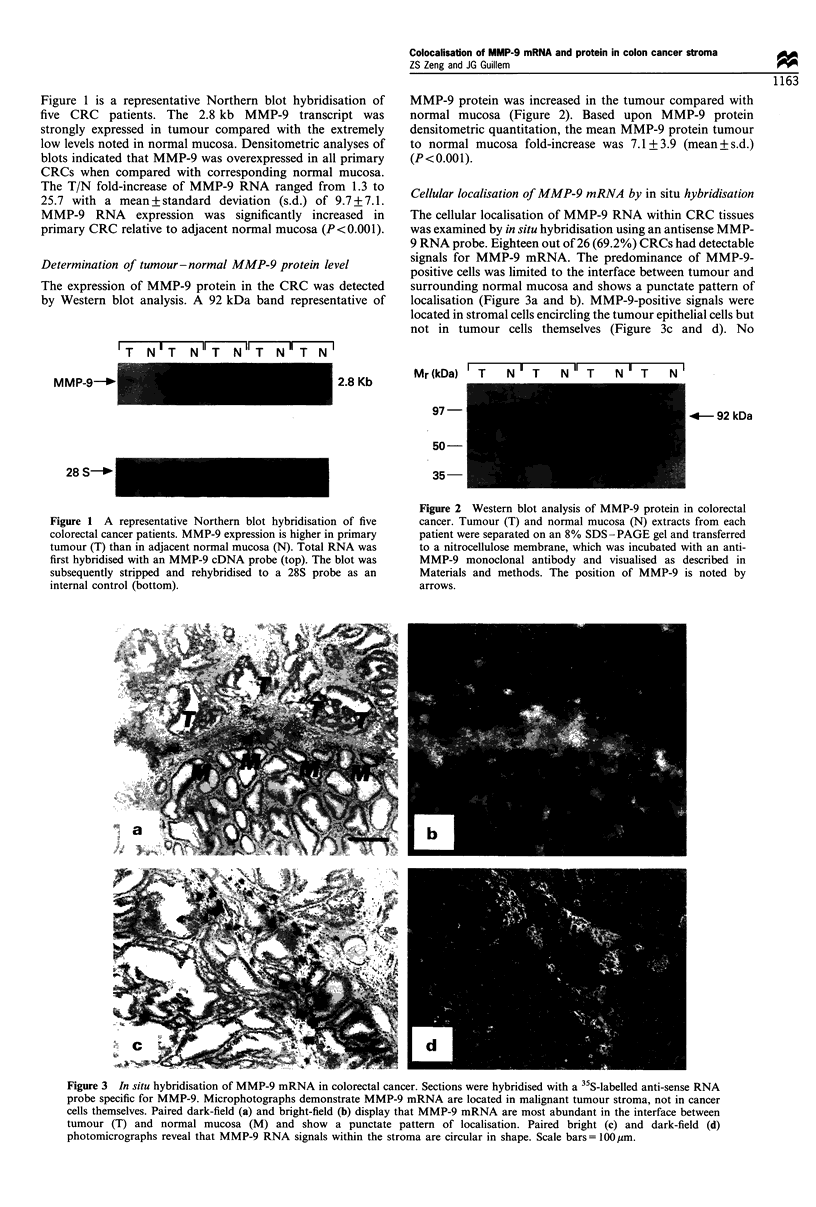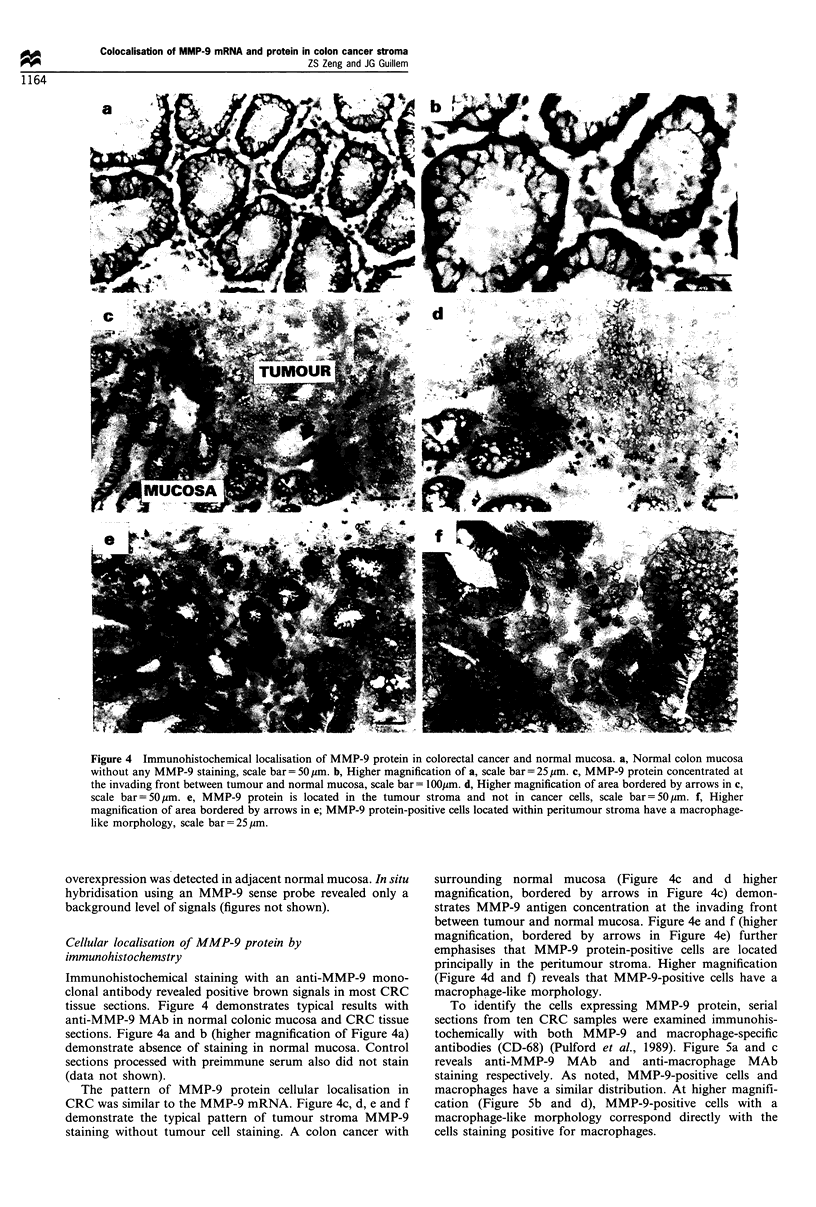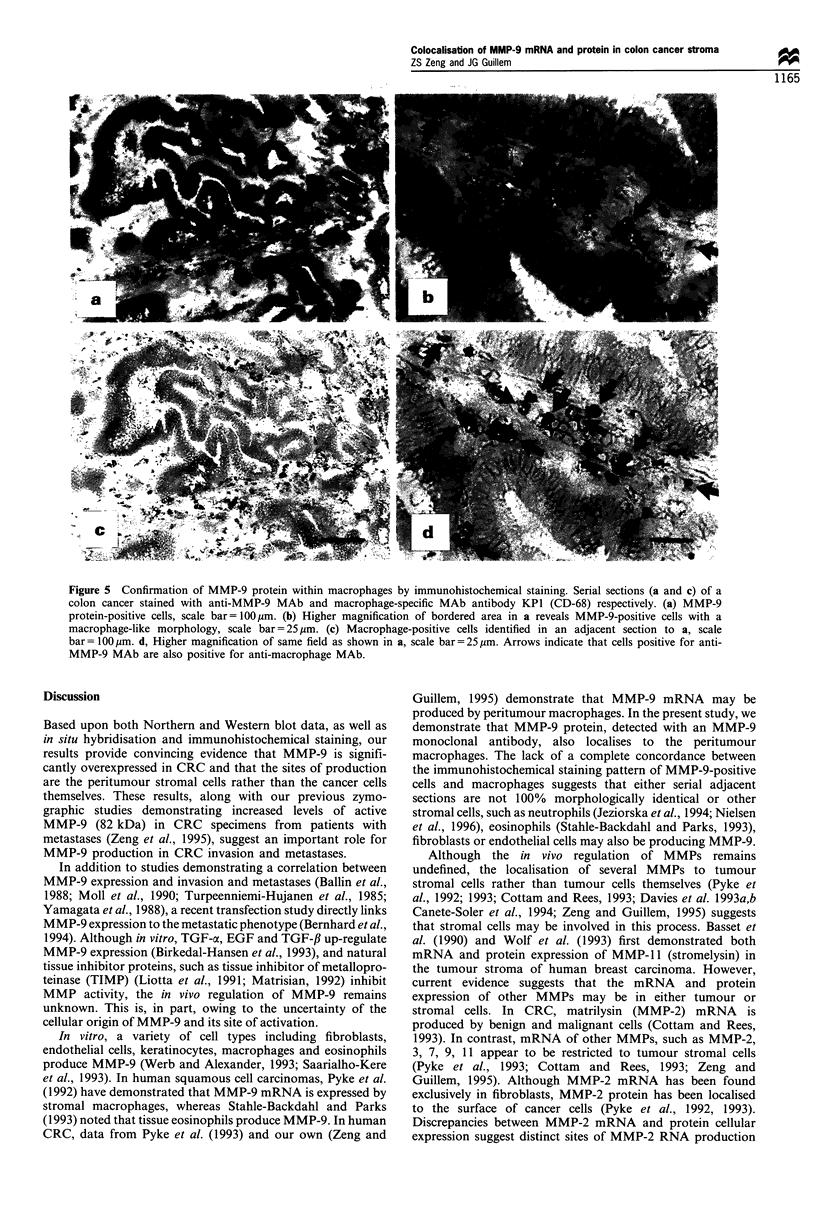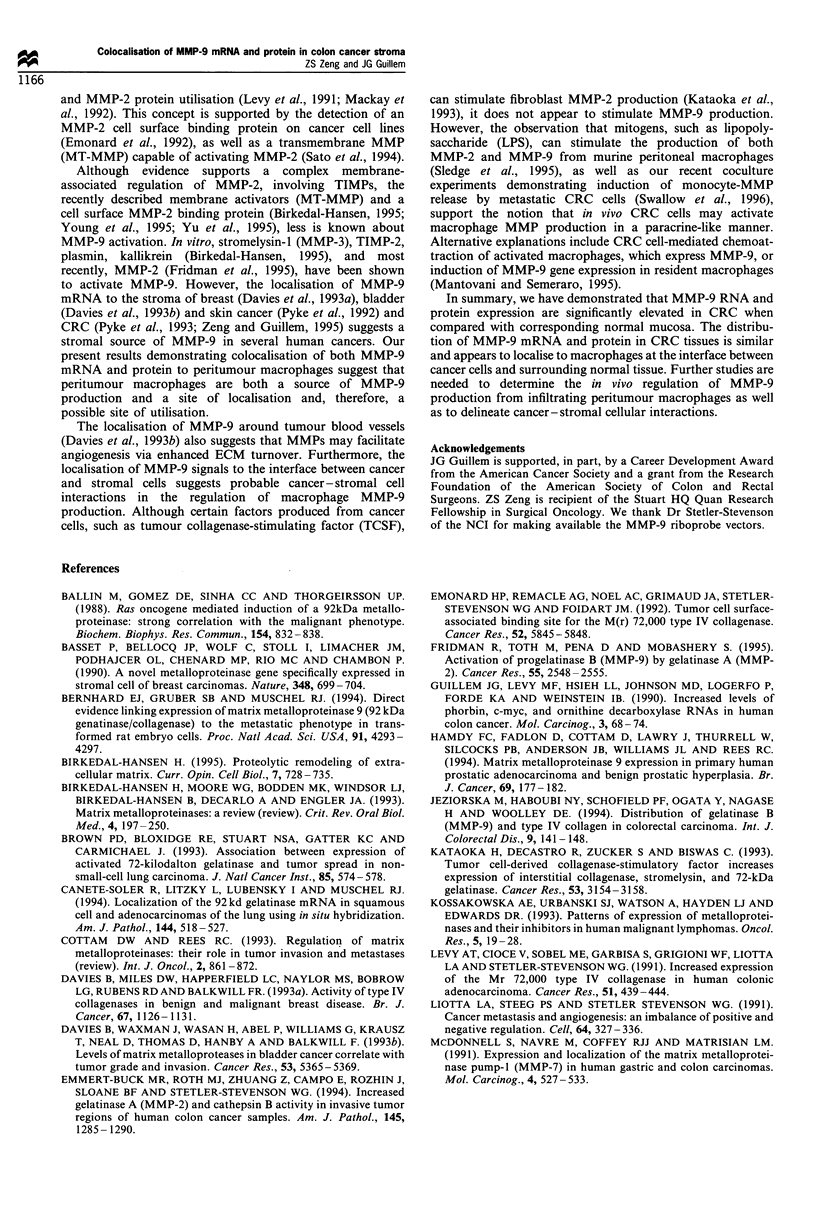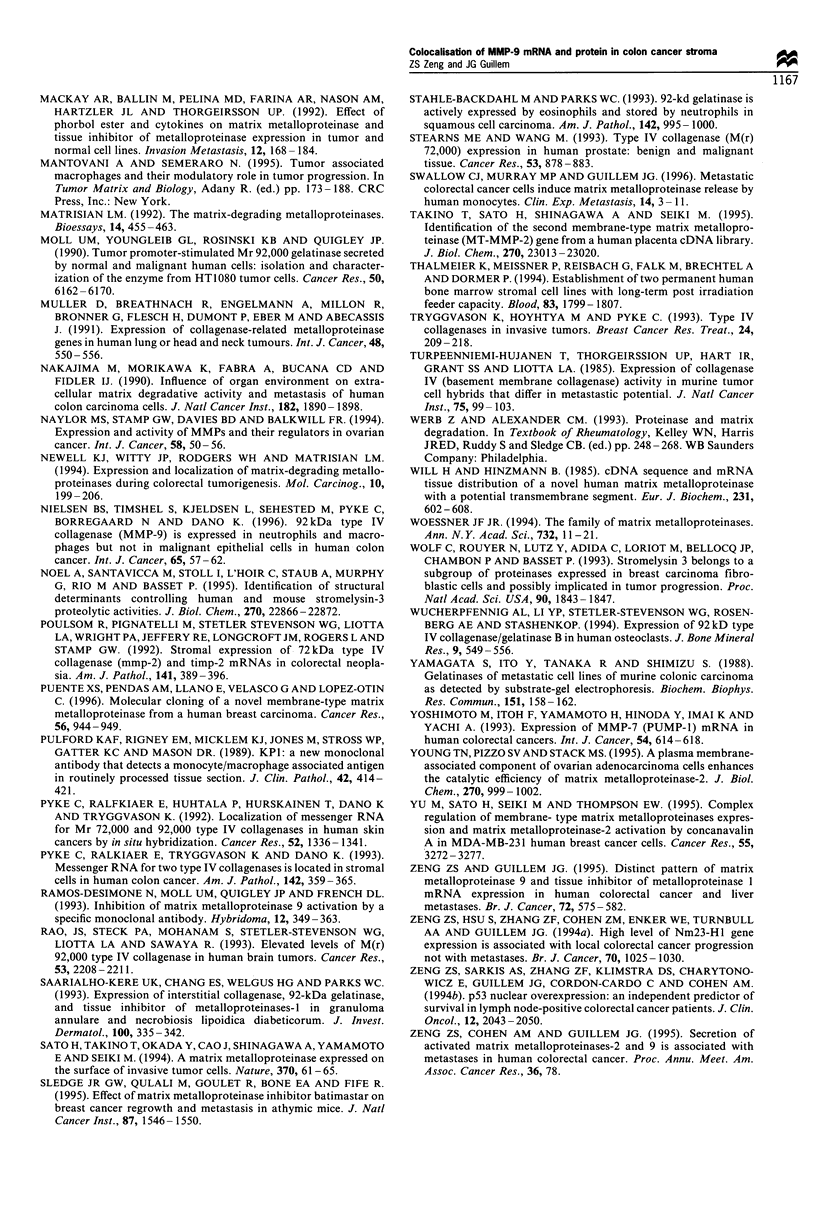# Colocalisation of matrix metalloproteinase-9-mRNA and protein in human colorectal cancer stromal cells.

**DOI:** 10.1038/bjc.1996.511

**Published:** 1996-10

**Authors:** Z. S. Zeng, J. G. Guillem

**Affiliations:** Department of Surgery, Memorial Sloan-Kettering Cancer Center, New York, USA.

## Abstract

**Images:**


British Journal of Cancer (1996) 74, 1161-1167
© 1996 Stockton Press All rights reserved 0007-0920/96 $12.00

### Colocalisation of matrix metalloproteinase-9-mRNA and protein in human colorectal cancer stromal cells

ZS Zeng and JG Guillem

*Colorectal Service, Department of Surgery, Memorial Sloan-Kettering Cancer Center, New York, USA.*

**Summary** The matrix metalloproteinases (MMPs) are perceived as essential for tumour invasion and metastases. The purpose of this study was to determine the expression and cellular localisation of the 92 kDa type IV collagenase (MMP-9) protein and mRNA in human colorectal cancer (CRC). In CRC and matched normal mucosa specimens from 26 CRC patients, Northern blot hybridisation and Western blot analyses provide convincing evidence that MMP-9 is expressed in greater quantities in CRC than in normal tissue. The MMP-9 tumour to normal mucosa fold-increase (T/N) was $9.7 \pm 7.1$ (mean$\pm$s.d.) ($P<0.001$) for RNA and $7.1 \pm 3.9$ ($P<0.001$) for protein. The sites of MMP-9 mRNA and protein synthesis were colocalised in tumour stroma by *in situ* hybridisation and immunohistochemistry in 26 CRC samples. Both MMP-9 mRNA and protein signals were strongest in the population of stromal cells concentrated at the tumour-stroma interface of an invading tumour. Furthermore, MMP-9-positive cells were identified as macrophages using an anti-macrophage antibody (KP1) in serial sections from ten CRC samples. Given the persistent localisation of MMP-9-producing macrophages to the interphase between CRC and surrounding stroma, our observations suggest that MMP-9 production is controlled, in part, by tumour-stroma cell interactions. Further studies are needed to determine the *in vivo* regulation of MMP-9 production from infiltrating peritumour macrophages.

**Keywords:** 92 type IV collagenase; matrix metalloproteinase; colorectal cancer; macrophage

Crucial steps in tumour invasion and metastases are the breaching of the basement membrane (BM) and degradation of the extracellular matrix (ECM) (Liotta *et al.*, 1991; Matrisian, 1992). These processes are likely to involve numerous proteolytic enzymes, including matrix metalloproteinases (MMPs), a family of extracellular matrix-degrading enzymes (Liotta *et al.*, 1991). To date, at least 14 members of the MMP family have been described by substrate specificity (Birkedal-Hansen, 1995). Based on substrate preference, MMPs can be subclassified into interstitial collagenases (MMP-1, MMP-8 and MMP-13), type IV collagenases/gelatinase (MMP-2 and MMP-9), stromelysins (MMP-3 and MMP-10), membrane-type MMPs [MT-MMP1 (Sato *et al.*, 1994), MT-MMP2 (Will and Hinzmann, 1995), MT-MMP3 (Takino *et al.*, 1995) and MT-MMP4 (Puente *et al.*, 1996)]. However, several MMPs including, stromelysin 3 (MMP-11) (Noel *et al.*, 1995) and macrophage metalloelastase (MMP-12) (Woessner, 1994; Birkedal-Hansen, 1995) do not appear to belong to any one group.

An important role for MMPs in invasion and metastases is supported by studies demonstrating a correlation between elevated MMP levels and the metastatic phenotype in cell cultures (Ballin *et al.*, 1988; Moll *et al.*, 1990; Turpeenniemi-Hujanen *et al.*, 1985; Yamagata *et al.*, 1988), animal models (Nakajima *et al.*, 1990) and several human tumours (Muller et *al.*, 1991; McDonnell *et al.*, 1991; Hamdy *et al.*, 1994; Levy *et al.*, 1991; Stearns and Wang, 1993; Rao *et al.*, 1993; Yoshimoto *et al.*, 1993; Kossakowska *et al.*, 1993; Brown *et al.*, 1993; Naylor *et al.*, 1994).

Since type IV collagen is a major component of BM, the 72 kDa (MMP-2) and 92 kDa (MMP-9) type IV collagenase are of particular interest. In order to understand further *in vivo* type IV collagenase regulation, knowledge of the *in vivo* sources of MMP-2 and MMP-9 production is essential. However, owing to conflicting reports localising MMP-2 mRNA to peritumour stromal fibroblasts (Poulsom *et al.*, 1992; Pyke *et al.*, 1993) and MMP-2 protein to CRC cells themselves (Levy *et al.*, 1991), the cellular origin of MMP-2 within CRC specimens remains unclear. Similarly, although microdissection studies on human CRC specimens localise increased pro-MMP-9 enzyme to the invasive CRC edge (Emmert-Buck *et al.*, 1994), the cellular source of MMP-9 remains uncertain, since MMP-9 mRNA signals have been localised to the peritumour stromal compartment of breast (Tryggvason *et al.*, 1993; Davies *et al.*, 1993*a*), skin (Pyke *et al.*, 1992), bladder (Davies *et al.*, 1993*b*) and colorectal cancers (Pyke *et al.*, 1993; Newell *et al.*, 1994; Zeng and Guillem, 1995), while MMP-9 immunostaining has been localised within CRC cells as well as peritumour stromal neutrophils and macrophages (Jeziorska *et al.*, 1994). Furthermore, in human osteoclastomas, both MMP-9 RNA and protein have been localised to multinucleated giant cells (Wucherpfennig *et al.*, 1994). In order to clarify the discrepancy in the cellular origin of CRC MMP-9, we examined the simultaneous mRNA and protein pattern of MMP-9 expression in human CRC specimens.

### Materials and methods

### *Tissue preparation*

Twenty-six colorectal cancers and paired normal tissue used in this study were obtained from the operating room immediately after resection with the approval of the Institutional Review Board of the Memorial Sloan-Kettering Cancer Center. They were quick frozen in liquid nitrogen and stored at $-80°C$ until processed. Samples were handled and stored under strict RNAase-free conditions. Frozen-section tissue was embedded in OCT (Miles, Elkhart, IN, USA) and frozen in 2-methylbutane cooled with liquid nitrogen. For *in situ* hybridisation, specimens were fixed in RNAase-free 4% paraformaldehyde overnight at 4°C, sequentially dehydrated with 50%, 70%, 85%, 95% and 100% ethanol and embedded in paraffin.

### *Northern blot hybridisation*

RNA was extracted by the guanidium isocyanide-caesium chloride methods, as previously described (Guillem *et al.*, 1990). Total RNA (10 $\mu$g) were electrophoresed on 1.0%

Correspondence: JG Guillem, Colorectal Service, MSKCC, 1275 York Avenue, NY 10021, USA
Received 19 January 1996; revised 12 April 1996; accepted 2 May 1996

agarose–formaldehyde gel and blotted onto a Duralon-UV membrane (Stratagene). MMP-9 DNA probes were radiolabelled with [$^{32}$P]dCTP by the random primer technique. A 28S oligo probe was used as an internal control for equal RNA loading and ethidium bromide staining of gels to confirm equal RNA transfer.

### In situ *hybridisation*

Our techique for MMP-9 *in situ* hybridisation has been previously described in detail (Zeng and Guillem, 1995). Briefly, the sense and antisense transcripts were prepared from a human MMP-9 cDNA insert (1059 bp extending from nucleotides 7 to 1066) subcloned in Bluescript KS (Stratagene). The MMP-9 sense probe was generated by $T_3$ polymerase following digestion with *Xba*I, while the antisense probe was generated by $T_7$ polymerase after *Eco*RI template digestion respectively. Transcribed RNA was labelled with α[$^{35}$S]UTP (1200 Ci mmol$^{-1}$, DuPont NEN). DNA template was removed by incubating with RNAase-free DNAase. tRNA (10 μg) was added and samples were extracted with phenol–chloroform. RNA probes were hydrolysed with sodium carbonate buffer, pH 10.2, for 60 min at 60°C, neutralised and ethanol precipitated.

Paraffin sections (5–10 μM thick) were dried, deparaffinised and redehydrated. Slides were incubated with proteinase K (100 μg ml$^{-1}$), washed in 2 mg ml$^{-1}$ glycine (w/v), and lastly, washed in triethanolamine buffer containing 0.25% acetic anhydride. Sections were covered with $^{35}$S-labelled RNA probe and sealed with a coverslip and incubated at 57°C overnight. After hybridisation, slides were washed in 4 × saline sodium citrate (SSC) for 1 h and then in 2 × SSC, 50% formamide, 10 mM DTT solution for 40 min at 68°C. Slides were then treated with 20 μg ml$^{-1}$ RNAase-A in 20 mM Tris, pH 7.5, 0.5 M sodium chloride, 1 mM EDTA at 37°C for 30 min, followed by washing in the same buffer without RNAase-A for 30 min. The final washes were in 2 × SSC, 50% formamide, 1 mM DTT for 40 min at 68°C, 2 × SSC for 5 min at room temperature and 0.1 × SSC for 15 min at 50°C. Slides were dehydrated through graded ethanol and air dried.

Autoradiographic detection of the hybrids was carried out by dipping in Kodak NTB-2 emulsion (Eastman Kodak, Rochester, NY, USA) at 42°C under safelight and dried. Slides were developed and fixed after 10–14 days exposure. Tissue sections were counterstained with haematoxylin and eosin (H&E). The sections were examined by dark-field microscopy.

### *Monoclonal antibody*

The monoclonal antibody (MAb) MMP-9 (Ab-2) (clone 7–11C) was obtained from Oncogene Science, Inc. (Manhassett, NY, USA). This MAb is generated by immunising mice with MMP-9 protein partially purified from the conditioned media of PMA-stimulated HT1080 human fibrosarcoma cells (Ramos-DeSimone *et al.*, 1993). MMP-9 (Ab-2) recognises the latent (92 kDa) form of human MMP-9. The active (82 kDa) form of human MMP-9 cannot be recognised by this antibody (Ramos-DeSimone *et al.*, 1993). MAb MMP-9 (Ab-2) was used for both Western blot and immunohistochemistry. The anti-human macrophage monoclonal antibody, CD-68 (KP-1), was obtained from Dako Corporation (Glostrup, Denmark). This antibody is known to react with macrophages in a wide variety of human tissues, including Kupffer's cells and macrophages in the red pulp of the spleen, in the lamina propria of the gut, in lung alveoli and in bone marrow (Pulford *et al.*, 1989). However, not all haematopoietic cells react with KP1 antibody (Thalmeier *et al.*, 1994).

### *Western blot analysis*

The tissue was homogenised in Tris buffer (50 mM Tris-HCl, pH 7.5, containing 75 mM sodium chloride) and centrifuged at 5000 × *g* for 20 min as previously described (Zeng *et al.*, 1994*a*). The supernatant of tumour and paired normal mucosa (25 μg) were electrophoresed on a 8% sodium dodecyl sulphate-polyacrylamide gel electrophoresis gel using a MINIGEL apparatus (Bio-Rad, Richmond, CA, USA). Separated proteins were transferred to nitrocellulose membranes (Amersham, Bucks, UK) in Tris/glycine buffer (2.5 mM Tris, 192 mM glycine and 20% methanol) at 4°C and 100 V using a MINI system. Non-specific binding sites were blocked for 1 h at room temperature in 10 mM Tris buffer containing 150 mM sodium chloride and 0.5% Tween 20 (TBS-T) with 4% bovine serum albumin (BSA). The blots were incubated overnight at 4°C in a 1:500 dilution of MMP-9 MAb Ab-2. The blot was washed several times with TBS-T, followed by an incubation step with horseradish peroxidase-labelled anti-rabbit antibody (1:5000 in TBS-T for 30 min at room temperature). After washing with TBS-T, an enhanced chemiluminescence detection system (ECL, Amersham) was used. For molecular weight determination, ECL protein molecular weight and rainbow coloured protein molecular weight markers (Amersham) were used.

### *Immunohistochemistry*

For immunohistochemical staining, the slides were processed by use of the Vectastain ABC Elite kits (Vector Laboratories, Inc., Burlingame, CA, USA) according to the manufacturer's protocol as previously described (Zeng *et al.*, 1994*b*). Immediately before staining, frozen sections were fixed in 4°C acetone then rinsed with phosphate-buffered saline (PBS).

For the reduction of non-specific background staining, slides were incubated with diluted normal blocking serum for 20 min at room temperature. The serum was drained off and sections were incubated with the MAb MMP-9 (Ab-2) at a concentration of 200 ng ml$^{-1}$ at 4°C overnight. After washing, the slides were incubated with diluted biotinylated secondary antibody solution for 30 min, rinsed with PBS and then incubated with Vectastain Elite ABC reagent for 30 min. Following this, tissue sections were rinsed in PBS and immunostaining was developed by immersion in 0.06% 3.3′-diaminobenzidine tetrahydrochloride (DAB) solution dissolved in 0.5% Triton-X/PBS for 4 min. Sections were counterstained with modified Horris-Haematoxylin (Fisher) and 0.3% ammonia water and passed through graded alcohols and xylene to dehydrate. Slides were then observed by conventional light microscopy.

Tissue sections adjacent to those used for MMP-9 staining were stained by a MAb KP-1 (CD-68).

### *Densitometric quantitation*

MMP-9 RNA and protein levels were quantitated by measuring the intensities of the appropriate autoradiographic bands using LKB XL laser densitometry (Pharmacia LKB Biotechnology, Uppsala, Sweden).

The RNA results were expressed as the fold-increase of a 2.8 kilobase MMP-9 transcript in tumours to that in the paired normal tissues. The 28S transcript was used as an internal control:

$$T/N \text{ (Tumour/Normal mucosa)} = T_{MMP\text{-}9}:T_{28S}/N_{MMP\text{-}9}:N_{28S}$$

The MMP-9 protein levels are expressed as the fold-increase in expression of the 92 kDa bands in tumour relative to that measured in the corresponding adjacent normal mucosa.

Differences of both RNA and protein levels between tumour and paired normal tissue were analysed by Student's *t*-test.

### Results

### *Determination of tumour–normal MMP-9 RNA level*

Total cellular RNA of human CRC and paired adjacent normal mucosa from 26 CRC patients was examined for expression of MMP-9 RNA by Northern blot hybridisation.

Figure 1 is a representative Northern blot hybridisation of five CRC patients. The 2.8 kb MMP-9 transcript was strongly expressed in tumour compared with the extremely low levels noted in normal mucosa. Densitometric analyses of blots indicated that MMP-9 was overexpressed in all primary CRCs when compared with corresponding normal mucosa. The T/N fold-increase of MMP-9 RNA ranged from 1.3 to 25.7 with a mean $\pm$ standard deviation (s.d.) of $9.7 \pm 7.1$. MMP-9 RNA expression was significantly increased in primary CRC relative to adjacent normal mucosa ($P < 0.001$).

*Determination of tumour–normal MMP-9 protein level*

The expression of MMP-9 protein in the CRC was detected by Western blot analysis. A 92 kDa band representative of MMP-9 protein was increased in the tumour compared with normal mucosa (Figure 2). Based upon MMP-9 protein densitometric quantitation, the mean MMP-9 protein tumour to normal mucosa fold-increase was $7.1 \pm 3.9$ (mean $\pm$ s.d.) ($P < 0.001$).

*Cellular localisation of MMP-9 mRNA by* in situ *hybridisation*

The cellular localisation of MMP-9 RNA within CRC tissues was examined by *in situ* hybridisation using an antisense MMP-9 RNA probe. Eighteen out of 26 (69.2%) CRCs had detectable signals for MMP-9 mRNA. The predominance of MMP-9-positive cells was limited to the interface between tumour and surrounding normal mucosa and shows a punctate pattern of localisation (Figure 3a and b). MMP-9-positive signals were located in stromal cells encircling the tumour epithelial cells but not in tumour cells themselves (Figure 3c and d). No

**Figure 1** A representative Northern blot hybridisation of five colorectal cancer patients. MMP-9 expression is higher in primary tumour (T) than in adjacent normal mucosa (N). Total RNA was first hybridised with an MMP-9 cDNA probe (top). The blot was subsequently stripped and rehybridised to a 28S probe as an internal control (bottom).

**Figure 2** Western blot analysis of MMP-9 protein in colorectal cancer. Tumour (T) and normal mucosa (N) extracts from each patient were separated on an 8% SDS–PAGE gel and transferred to a nitrocellulose membrane, which was incubated with an anti-MMP-9 monoclonal antibody and visualised as described in Materials and methods. The position of MMP-9 is noted by arrows.

**Figure 3** *In situ* hybridisation of MMP-9 mRNA in colorectal cancer. Sections were hybridised with a $^{35}S$-labelled anti-sense RNA probe specific for MMP-9. Microphotographs demonstrate MMP-9 mRNA are located in malignant tumour stroma, not in cancer cells themselves. Paired dark-field (**a**) and bright-field (**b**) display that MMP-9 mRNA are most abundant in the interface between tumour (T) and normal mucosa (M) and show a punctate pattern of localisation. Paired bright (**c**) and dark-field (**d**) photomicrographs reveal that MMP-9 RNA signals within the stroma are circular in shape. Scale bars = 100 $\mu$m.

**Figure 4** Immunohistochemical localisation of MMP-9 protein in colorectal cancer and normal mucosa. **a**, Normal colon mucosa without any MMP-9 staining, scale bar = 50 $\mu$m. **b**, Higher magnification of **a**, scale bar = 25 $\mu$m. **c**, MMP-9 protein concentrated at the invading front between tumour and normal mucosa, scale bar = 100$\mu$m. **d**, Higher magnification of area bordered by arrows in **c**, scale bar = 50 $\mu$m. **e**, MMP-9 protein is located in the tumour stroma and not in cancer cells, scale bar = 50 $\mu$m. **f**, Higher magnification of area bordered by arrows in **e**; MMP-9 protein-positive cells located within peritumour stroma have a macrophage-like morphology, scale bar = 25 $\mu$m.

overexpression was detected in adjacent normal mucosa. *In situ* hybridisation using an MMP-9 sense probe revealed only a background level of signals (figures not shown).

*Cellular localisation of MMP-9 protein by immunohistochemstry*

Immunohistochemical staining with an anti-MMP-9 monoclonal antibody revealed positive brown signals in most CRC tissue sections. Figure 4 demonstrates typical results with anti-MMP-9 MAb in normal colonic mucosa and CRC tissue sections. Figure 4a and b (higher magnification of Figure 4a) demonstrate absence of staining in normal mucosa. Control sections processed with preimmune serum also did not stain (data not shown).

The pattern of MMP-9 protein cellular localisation in CRC was similar to the MMP-9 mRNA. Figure 4c, d, e and f demonstrate the typical pattern of tumour stroma MMP-9 staining without tumour cell staining. A colon cancer with surrounding normal mucosa (Figure 4c and d higher magnification, bordered by arrows in Figure 4c) demonstrates MMP-9 antigen concentration at the invading front between tumour and normal mucosa. Figure 4e and f (higher magnification, bordered by arrows in Figure 4e) further emphasises that MMP-9 protein-positive cells are located principally in the peritumour stroma. Higher magnification (Figure 4d and f) reveals that MMP-9-positive cells have a macrophage-like morphology.

To identify the cells expressing MMP-9 protein, serial sections from ten CRC samples were examined immunohistochemically with both MMP-9 and macrophage-specific antibodies (CD-68) (Pulford *et al.*, 1989). Figure 5a and c reveals anti-MMP-9 MAb and anti-macrophage MAb staining respectively. As noted, MMP-9-positive cells and macrophages have a similar distribution. At higher magnification (Figure 5b and d), MMP-9-positive cells with a macrophage-like morphology correspond directly with the cells staining positive for macrophages.

**Figure 5** Confirmation of MMP-9 protein within macrophages by immunohistochemical staining. Serial sections (**a** and **c**) of a colon cancer stained with anti-MMP-9 MAb and macrophage-specific MAb antibody KP1 (CD-68) respectively. (**a**) MMP-9 protein-positive cells, scale bar = 100 $\mu$m. (**b**) Higher magnification of bordered area in **a** reveals MMP-9-positive cells with a macrophage-like morphology, scale bar = 25 $\mu$m. (**c**) Macrophage-positive cells identified in an adjacent section to **a**, scale bar = 100 $\mu$m. **d**, Higher magnification of same field as shown in **a**, scale bar = 25 $\mu$m. Arrows indicate that cells positive for anti-MMP-9 MAb are also positive for anti-macrophage MAb.

### Discussion

Based upon both Northern and Western blot data, as well as *in situ* hybridisation and immunohistochemical staining, our results provide convincing evidence that MMP-9 is significantly overexpressed in CRC and that the sites of production are the peritumour stromal cells rather than the cancer cells themselves. These results, along with our previous zymographic studies demonstrating increased levels of active MMP-9 (82 kDa) in CRC specimens from patients with metastases (Zeng *et al.*, 1995), suggest an important role for MMP-9 production in CRC invasion and metastases.

In addition to studies demonstrating a correlation between MMP-9 expression and invasion and metastases (Ballin *et al.*, 1988; Moll *et al.*, 1990; Turpeenniemi-Hujanen *et al.*, 1985; Yamagata *et al.*, 1988), a recent transfection study directly links MMP-9 expression to the metastatic phenotype (Bernhard *et al.*, 1994). Although *in vitro*, TGF-$\alpha$, EGF and TGF-$\beta$ up-regulate MMP-9 expression (Birkedal-Hansen *et al.*, 1993), and natural tissue inhibitor proteins, such as tissue inhibitor of metalloproteinase (TIMP) (Liotta *et al.*, 1991; Matrisian, 1992) inhibit MMP activity, the *in vivo* regulation of MMP-9 remains unknown. This is, in part, owing to the uncertainty of the cellular origin of MMP-9 and its site of activation.

*In vitro*, a variety of cell types including fibroblasts, endothelial cells, keratinocytes, macrophages and eosinophils produce MMP-9 (Werb and Alexander, 1993; Saarialho-Kere *et al.*, 1993). In human squamous cell carcinomas, Pyke *et al.* (1992) have demonstrated that MMP-9 mRNA is expressed by stromal macrophages, whereas Stahle-Backdahl and Parks (1993) noted that tissue eosinophils produce MMP-9. In human CRC, data from Pyke *et al.* (1993) and our own (Zeng and Guillem, 1995) demonstrate that MMP-9 mRNA may be produced by peritumour macrophages. In the present study, we demonstrate that MMP-9 protein, detected with an MMP-9 monoclonal antibody, also localises to the peritumour macrophages. The lack of a complete concordance between the immunohistochemical staining pattern of MMP-9-positive cells and macrophages suggests that either serial adjacent sections are not 100% morphologically identical or other stromal cells, such as neutrophils (Jeziorska *et al.*, 1994; Nielsen *et al.*, 1996), eosinophils (Stahle-Backdahl and Parks, 1993), fibroblasts or endothelial cells may also be producing MMP-9.

Although the *in vivo* regulation of MMPs remains undefined, the localisation of several MMPs to tumour stromal cells rather than tumour cells themselves (Pyke *et al.*, 1992; 1993; Cottam and Rees, 1993; Davies *et al.* 1993*a,b* Canete-Soler *et al.*, 1994; Zeng and Guillem, 1995) suggests that stromal cells may be involved in this process. Basset *et al.* (1990) and Wolf *et al.* (1993) first demonstrated both mRNA and protein expression of MMP-11 (stromelysin) in the tumour stroma of human breast carcinoma. However, current evidence suggests that the mRNA and protein expression of other MMPs may be in either tumour or stromal cells. In CRC, matrilysin (MMP-2) mRNA is produced by benign and malignant cells (Cottam and Rees, 1993). In contrast, mRNA of other MMPs, such as MMP-2, 3, 7, 9, 11 appear to be restricted to tumour stromal cells (Pyke *et al.*, 1993; Cottam and Rees, 1993; Zeng and Guillem, 1995). Although MMP-2 mRNA has been found exclusively in fibroblasts, MMP-2 protein has been localised to the surface of cancer cells (Pyke *et al.*, 1992, 1993). Discrepancies between MMP-2 mRNA and protein cellular expression suggest distinct sites of MMP-2 RNA production and MMP-2 protein utilisation (Levy *et al.*, 1991; Mackay *et al.*, 1992). This concept is supported by the detection of an MMP-2 cell surface binding protein on cancer cell lines (Emonard *et al.*, 1992), as well as a transmembrane MMP (MT-MMP) capable of activating MMP-2 (Sato *et al.*, 1994).

Although evidence supports a complex membrane-associated regulation of MMP-2, involving TIMPs, the recently described membrane activators (MT-MMP) and a cell surface MMP-2 binding protein (Birkedal-Hansen, 1995; Young *et al.*, 1995; Yu *et al.*, 1995), less is known about MMP-9 activation. *In vitro*, stromelysin-1 (MMP-3), TIMP-2, plasmin, kallikrein (Birkedal-Hansen, 1995), and most recently, MMP-2 (Fridman *et al.*, 1995), have been shown to activate MMP-9. However, the localisation of MMP-9 mRNA to the stroma of breast (Davies *et al.*, 1993*a*), bladder (Davies *et al.*, 1993*b*) and skin cancer (Pyke *et al.*, 1992) and CRC (Pyke *et al.*, 1993; Zeng and Guillem, 1995) suggests a stromal source of MMP-9 in several human cancers. Our present results demonstrating colocalisation of both MMP-9 mRNA and protein to peritumour macrophages suggest that peritumour macrophages are both a source of MMP-9 production and a site of localisation and, therefore, a possible site of utilisation.

The localisation of MMP-9 around tumour blood vessels (Davies et al., 1993*b*) also suggests that MMPs may facilitate angiogenesis via enhanced ECM turnover. Furthermore, the localisation of MMP-9 signals to the interface between cancer and stromal cells suggests probable cancer–stromal cell interactions in the regulation of macrophage MMP-9 production. Although certain factors produced from cancer cells, such as tumour collagenase-stimulating factor (TCSF), can stimulate fibroblast MMP-2 production (Kataoka *et al.*, 1993), it does not appear to stimulate MMP-9 production. However, the observation that mitogens, such as lipopolysaccharide (LPS), can stimulate the production of both MMP-2 and MMP-9 from murine peritoneal macrophages (Sledge *et al.*, 1995), as well as our recent coculture experiments demonstrating induction of monocyte-MMP release by metastatic CRC cells (Swallow *et al.*, 1996), support the notion that *in vivo* CRC cells may activate macrophage MMP production in a paracrine-like manner. Alternative explanations include CRC cell-mediated chemoattraction of activated macrophages, which express MMP-9, or induction of MMP-9 gene expression in resident macrophages (Mantovani and Semeraro, 1995).

In summary, we have demonstrated that MMP-9 RNA and protein expression are significantly elevated in CRC when compared with corresponding normal mucosa. The distribution of MMP-9 mRNA and protein in CRC tissues is similar and appears to localise to macrophages at the interface between cancer cells and surrounding normal tissue. Further studies are needed to determine the *in vivo* regulation of MMP-9 production from infiltrating peritumour macrophages as well as to delineate cancer–stromal cellular interactions.

**Acknowledgements**
JG Guillem is supported, in part, by a Career Development Award from the American Cancer Society and a grant from the Research Foundation of the American Society of Colon and Rectal Surgeons. ZS Zeng is recipient of the Stuart HQ Quan Research Fellowship in Surgical Oncology. We thank Dr Stetler-Stevenson of the NCI for making available the MMP-9 riboprobe vectors.

### References

BALLIN M, GOMEZ DE, SINHA CC AND THORGEIRSSON UP. (1988). *Ras* oncogene mediated induction of a 92kDa metalloproteinase: strong correlation with the malignant phenotype. *Biochem. Biophys. Res. Commun.*, **154**, 832–838.

BASSET P, BELLOCQ JP, WOLF C, STOLL I, LIMACHER JM, PODHAJCER OL, CHENARD MP, RIO MC AND CHAMBON P. (1990). A novel metalloproteinase gene specifically expressed in stromal cell of breast carcinomas. *Nature*, **348**, 699–704.

BERNHARD EJ, GRUBER SB AND MUSCHEL RJ. (1994). Direct evidence linking expression of matrix metalloproteinase 9 (92 kDa genatinase/collagenase) to the metastatic phenotype in transformed rat embryo cells. *Proc. Natl Acad. Sci. USA*, **91**, 4293–4297.

BIRKEDAL-HANSEN H. (1995). Proteolytic remodeling of extracellular matrix. *Curr. Opin. Cell Biol.*, **7**, 728–735.

BIRKEDAL-HANSEN H, MOORE WG, BODDEN MK, WINDSOR LJ, BIRKEDAL-HANSEN B, DECARLO A AND ENGLER JA. (1993). Matrix metalloproteinases: a review (review). *Crit. Rev. Oral Biol. Med.*, **4**, 197–250.

BROWN PD, BLOXIDGE RE, STUART NSA, GATTER KC AND CARMICHAEL J. (1993). Association between expression of activated 72-kilodalton gelatinase and tumor spread in non-small-cell lung carcinoma. *J. Natl Cancer Inst.*, **85**, 574–578.

CANETE-SOLER R, LITZKY L, LUBENSKY I AND MUSCHEL RJ. (1994). Localization of the 92 kd gelatinase mRNA in squamous cell and adenocarcinomas of the lung using *in situ* hybridization. *Am. J. Pathol.*, **144**, 518–527.

COTTAM DW AND REES RC. (1993). Regulation of matrix metalloproteinases: their role in tumor invasion and metastases (review). *Int. J. Oncol.*, **2**, 861–872.

DAVIES B, MILES DW, HAPPERFIELD LC, NAYLOR MS, BOBROW LG, RUBENS RD AND BALKWILL FR. (1993*a*). Activity of type IV collagenases in benign and malignant breast disease. *Br. J. Cancer*, **67**, 1126–1131.

DAVIES B, WAXMAN J, WASAN H, ABEL P, WILLIAMS G, KRAUSZ T, NEAL D, THOMAS D, HANBY A AND BALKWILL F. (1993*b*). Levels of matrix metalloproteases in bladder cancer correlate with tumor grade and invasion. *Cancer Res.*, **53**, 5365–5369.

EMMERT-BUCK MR, ROTH MJ, ZHUANG Z, CAMPO E, ROZHIN J, SLOANE BF AND STETLER-STEVENSON WG. (1994). Increased gelatinase A (MMP-2) and cathepsin B activity in invasive tumor regions of human colon cancer samples. *Am. J. Pathol.*, **145**, 1285–1290.

EMONARD HP, REMACLE AG, NOEL AC, GRIMAUD JA, STETLER-STEVENSON WG AND FOIDART JM. (1992). Tumor cell surface-associated binding site for the M(r) 72,000 type IV collagenase. *Cancer Res.*, **52**, 5845–5848.

FRIDMAN R, TOTH M, PENA D AND MOBASHERY S. (1995). Activation of progelatinase B (MMP-9) by gelatinase A (MMP-2). *Cancer Res.*, **55**, 2548–2555.

GUILLEM JG, LEVY MF, HSIEH LL, JOHNSON MD, LOGERFO P, FORDE KA AND WEINSTEIN IB. (1990). Increased levels of phorbin, c-myc, and ornithine decarboxylase RNAs in human colon cancer. *Mol. Carcinog.*, **3**, 68–74.

HAMDY FC, FADLON D, COTTAM D, LAWRY J, THURRELL W, SILCOCKS PB, ANDERSON JB, WILLIAMS JL AND REES RC. (1994). Matrix metalloproteinase 9 expression in primary human prostatic adenocarcinoma and benign prostatic hyperplasia. *Br. J. Cancer*, **69**, 177–182.

JEZIORSKA M, HABOUBI NY, SCHOFIELD PF, OGATA Y, NAGASE H AND WOOLLEY DE. (1994). Distribution of gelatinase B (MMP-9) and type IV collagen in colorectal carcinoma. *Int. J. Colorectal Dis.*, **9**, 141–148.

KATAOKA H, DECASTRO R, ZUCKER S AND BISWAS C. (1993). Tumor cell-derived collagenase-stimulatory factor increases expression of interstitial collagenase, stromelysin, and 72-kDa gelatinase. *Cancer Res.*, **53**, 3154–3158.

KOSSAKOWSKA AE, URBANSKI SJ, WATSON A, HAYDEN LJ AND EDWARDS DR. (1993). Patterns of expression of metalloproteinases and their inhibitors in human malignant lymphomas. *Oncol. Res.*, **5**, 19–28.

LEVY AT, CIOCE V, SOBEL ME, GARBISA S, GRIGIONI WF, LIOTTA LA AND STETLER-STEVENSON WG. (1991). Increased expression of the Mr 72,000 type IV collagenase in human colonic adenocarcinoma. *Cancer Res.*, **51**, 439–444.

LIOTTA LA, STEEG PS AND STETLER STEVENSON WG. (1991). Cancer metastasis and angiogenesis: an imbalance of positive and negative regulation. *Cell*, **64**, 327–336.

MCDONNELL S, NAVRE M, COFFEY RJJ AND MATRISIAN LM. (1991). Expression and localization of the matrix metalloproteinase pump-1 (MMP-7) in human gastric and colon carcinomas. *Mol. Carcinog.*, **4**, 527–533.

MACKAY AR, BALLIN M, PELINA MD, FARINA AR, NASON AM, HARTZLER JL AND THORGEIRSSON UP. (1992). Effect of phorbol ester and cytokines on matrix metalloproteinase and tissue inhibitor of metalloproteinase expression in tumor and normal cell lines. *Invasion Metastasis*, **12,** 168–184.
MANTOVANI A AND SEMERARO N. (1995). Tumor associated macrophages and their modulatory role in tumor progression. In *Tumor Matrix and Biology*, Adany R. (ed.) pp. 173–188. CRC Press, Inc.: New York.
MATRISIAN LM. (1992). The matrix-degrading metalloproteinases. *Bioessays*, **14,** 455–463.
MOLL UM, YOUNGLEIB GL, ROSINSKI KB AND QUIGLEY JP. (1990). Tumor promoter-stimulated Mr 92,000 gelatinase secreted by normal and malignant human cells: isolation and characterization of the enzyme from HT1080 tumor cells. *Cancer Res.*, **50,** 6162–6170.
MULLER D, BREATHNACH R, ENGELMANN A, MILLON R, BRONNER G, FLESCH H, DUMONT P, EBER M AND ABECASSIS J. (1991). Expression of collagenase-related metalloproteinase genes in human lung or head and neck tumours. *Int. J. Cancer*, **48,** 550–556.
NAKAJIMA M, MORIKAWA K, FABRA A, BUCANA CD AND FIDLER IJ. (1990). Influence of organ environment on extracellular matrix degradative activity and metastasis of human colon carcinoma cells. *J. Natl Cancer Inst.*, **182,** 1890–1898.
NAYLOR MS, STAMP GW, DAVIES BD AND BALKWILL FR. (1994). Expression and activity of MMPs and their regulators in ovarian cancer. *Int. J. Cancer*, **58,** 50–56.
NEWELL KJ, WITTY JP, RODGERS WH AND MATRISIAN LM. (1994). Expression and localization of matrix-degrading metalloproteinases during colorectal tumorigenesis. *Mol. Carcinog.*, **10,** 199–206.
NIELSEN BS, TIMSHEL S, KJELDSEN L, SEHESTED M, PYKE C, BORREGAARD N AND DANO K. (1996). 92 kDa type IV collagenase (MMP-9) is expressed in neutrophils and macrophages but not in malignant epithelial cells in human colon cancer. *Int. J. Cancer*, **65,** 57–62.
NOEL A, SANTAVICCA M, STOLL I, L'HOIR C, STAUB A, MURPHY G, RIO M AND BASSET P. (1995). Identification of structural determinants controlling human and mouse stromelysin-3 proteolytic activities. *J. Biol. Chem.*, **270,** 22866–22872.
POULSOM R, PIGNATELLI M, STETLER STEVENSON WG, LIOTTA LA, WRIGHT PA, JEFFERY RE, LONGCROFT JM, ROGERS L AND STAMP GW. (1992). Stromal expression of 72 kDa type IV collagenase (mmp-2) and timp-2 mRNAs in colorectal neoplasia. *Am. J. Pathol.*, **141,** 389–396.
PUENTE XS, PENDAS AM, LLANO E, VELASCO G AND LOPEZ-OTIN C. (1996). Molecular cloning of a novel membrane-type matrix metalloproteinase from a human breast carcinoma. *Cancer Res.*, **56,** 944–949.
PULFORD KAF, RIGNEY EM, MICKLEM KJ, JONES M, STROSS WP, GATTER KC AND MASON DR. (1989). KP1: a new monoclonal antibody that detects a monocyte/macrophage associated antigen in routinely processed tissue section. *J. Clin. Pathol.*, **42,** 414–421.
PYKE C, RALFKIAER E, HUHTALA P, HURSKAINEN T, DANO K AND TRYGGVASON K. (1992). Localization of messenger RNA for Mr 72,000 and 92,000 type IV collagenases in human skin cancers by *in situ* hybridization. *Cancer Res.*, **52,** 1336–1341.
PYKE C, RALKIAER E, TRYGGVASON K AND DANO K. (1993). Messenger RNA for two type IV collagenases is located in stromal cells in human colon cancer. *Am. J. Pathol.*, **142,** 359–365.
RAMOS-DESIMONE N, MOLL UM, QUIGLEY JP AND FRENCH DL. (1993). Inhibition of matrix metalloproteinase 9 activation by a specific monoclonal antibody. *Hybridoma*, **12,** 349–363.
RAO, JS, STECK PA, MOHANAM S, STETLER-STEVENSON WG, LIOTTA LA AND SAWAYA R. (1993). Elevated levels of M(r) 92,000 type IV collagenase in human brain tumors. *Cancer Res.*, **53,** 2208–2211.
SAARIALHO-KERE UK, CHANG ES, WELGUS HG AND PARKS WC. (1993). Expression of interstitial collagenase, 92-kDa gelatinase, and tissue inhibitor of metalloproteinases-1 in granuloma annulare and necrobiosis lipoidica diabeticorum. *J. Invest. Dermatol.*, **100,** 335–342.
SATO H, TAKINO T, OKADA Y, CAO J, SHINAGAWA A, YAMAMOTO E AND SEIKI M. (1994). A matrix metalloproteinase expressed on the surface of invasive tumor cells. *Nature*, **370,** 61–65.
SLEDGE JR GW, QULALI M, GOULET R, BONE EA AND FIFE R. (1995). Effect of matrix metalloproteinase inhibitor batimastar on breast cancer regrowth and metastasis in athymic mice. *J. Natl Cancer Inst.*, **87,** 1546–1550.
STAHLE-BACKDAHL M AND PARKS WC. (1993). 92-kd gelatinase is actively expressed by eosinophils and stored by neutrophils in squamous cell carcinoma. *Am. J. Pathol.*, **142,** 995–1000.
STEARNS ME AND WANG M. (1993). Type IV collagenase (M(r) 72,000) expression in human prostate: benign and malignant tissue. *Cancer Res.*, **53,** 878–883.
SWALLOW CJ, MURRAY MP AND GUILLEM JG. (1996). Metastatic colorectal cancer cells induce matrix metalloproteinase release by human monocytes. *Clin. Exp. Metastasis*, **14,** 3–11.
TAKINO T, SATO H, SHINAGAWA A AND SEIKI M. (1995). Identification of the second membrane-type matrix metalloproteinase (MT-MMP-2) gene from a human placenta cDNA library. *J. Biol. Chem.*, **270,** 23013–23020.
THALMEIER K, MEISSNER P, REISBACH G, FALK M, BRECHTEL A AND DORMER P. (1994). Establishment of two permanent human bone marrow stromal cell lines with long-term post irradiation feeder capacity. *Blood*, **83,** 1799–1807.
TRYGGVASON K, HOYHTYA M AND PYKE C. (1993). Type IV collagenases in invasive tumors. *Breast Cancer Res. Treat.*, **24,** 209–218.
TURPEENNIEMI-HUJANEN T, THORGEIRSSION UP, HART IR, GRANT SS AND LIOTTA LA. (1985). Expression of collagenase IV (basement membrane collagenase) activity in murine tumor cell hybrids that differ in metastastic potential. *J. Natl Cancer Inst.*, **75,** 99–103.
WERB Z AND ALEXANDER CM. (1993). Proteinase and matrix degradation. In *Textbook of Rheumatology*, Kelley WN, Harris JRED, Ruddy S and Sledge CB. (ed.) pp. 248–268. WB Saunders Company: Philadelphia.
WILL H AND HINZMANN B. (1985). cDNA sequence and mRNA tissue distribution of a novel human matrix metalloproteinase with a potential transmembrane segment. *Eur. J. Biochem.*, **231,** 602–608.
WOESSNER JF JR. (1994). The family of matrix metalloproteinases. *Ann. N.Y. Acad. Sci.*, **732,** 11–21.
WOLF C, ROUYER N, LUTZ Y, ADIDA C, LORIOT M, BELLOCQ JP, CHAMBON P AND BASSET P. (1993). Stromelysin 3 belongs to a subgroup of proteinases expressed in breast carcinoma fibroblastic cells and possibly implicated in tumor progression. *Proc. Natl Acad. Sci. USA*, **90,** 1843–1847.
WUCHERPFENNIG AL, LI YP, STETLER-STEVENSON WG, ROSENBERG AE AND STASHENKOP. (1994). Expression of 92 kD type IV collagenase/gelatinase B in human osteoclasts. *J. Bone Mineral Res.*, **9,** 549–556.
YAMAGATA S, ITO Y, TANAKA R AND SHIMIZU S. (1988). Gelatinases of metastatic cell lines of murine colonic carcinoma as detected by substrate-gel electrophoresis. *Biochem. Biophys. Res. Commun.*, **151,** 158–162.
YOSHIMOTO M, ITOH F, YAMAMOTO H, HINODA Y, IMAI K AND YACHI A. (1993). Expression of MMP-7 (PUMP-1) mRNA in human colorectal cancers. *Int. J. Cancer*, **54,** 614–618.
YOUNG TN, PIZZO SV AND STACK MS. (1995). A plasma membrane-associated component of ovarian adenocarcinoma cells enhances the catalytic efficiency of matrix metalloproteinase-2. *J. Biol. Chem.*, **270,** 999–1002.
YU M, SATO H, SEIKI M AND THOMPSON EW. (1995). Complex regulation of membrane- type matrix metalloproteinases expression and matrix metalloproteinase-2 activation by concanavalin A in MDA-MB-231 human breast cancer cells. *Cancer Res.*, **55,** 3272–3277.
ZENG ZS AND GUILLEM JG. (1995). Distinct pattern of matrix metalloproteinase 9 and tissue inhibitor of metalloproteinase 1 mRNA expression in human colorectal cancer and liver metastases. *Br. J. Cancer*, **72,** 575–582.
ZENG ZS, HSU S, ZHANG ZF, COHEN ZM, ENKER WE, TURNBULL AA AND GUILLEM JG. (1994*a*). High level of Nm23-H1 gene expression is associated with local colorectal cancer progression not with metastases. *Br. J. Cancer*, **70,** 1025–1030.
ZENG ZS, SARKIS AS, ZHANG ZF, KLIMSTRA DS, CHARYTONOWICZ E, GUILLEM JG, CORDON-CARDO C AND COHEN AM. (1994*b*). p53 nuclear overexpression: an independent predictor of survival in lymph node-positive colorectal cancer patients. *J. Clin. Oncol.*, **12,** 2043–2050.
ZENG ZS, COHEN AM AND GUILLEM JG. (1995). Secretion of activated matrix metalloproteinases-2 and 9 is associated with metastases in human colorectal cancer. *Proc. Annu. Meet. Am. Assoc. Cancer Res.*, **36,** 78.